# Dynamic stability evaluation of trunk accelerations during walking in blind and sighted individuals

**DOI:** 10.1186/s12886-024-03394-w

**Published:** 2024-03-21

**Authors:** Zeinab Rasouli Kahaki, Alireza Choobineh, Mohsen Razeghi, Mohammad Taghi Karimi, Ali Reza Safarpour

**Affiliations:** 1 Student Research Committee, Department of Ergonomics, School of Health, Shiraz University of Medical Sciences, Shiraz, Iran; 2 Research Center for Health Sciences, Institute of Health, Shiraz University of Medical Sciences, Shiraz, Iran; 3 Department of physiotherapy, School of Rehabilitation Sciences, Shiraz University of Medical Sciences, Shiraz, Iran; 4 Department of Orthotics and Prosthetics, School of Rehabilitation Sciences, Shiraz University of Medical Sciences, Shiraz, Iran; 5 Gastroenterohepatology Research Center, Shiraz University of Medical Sciences, Shiraz, Iran

**Keywords:** Blind, Dynamic stability, Harmonic ratio, Improved harmonic ratio, Root mean square

## Abstract

**Background:**

Dynamic stability is a fundamental goal in standing activities. In this regard, monitoring, analysis, and interventions made to improve stability is a research topic investigated in the biomechanics of human movements. Vision has a major role to play in controlling human movement. Nonetheless, little is known about the effects of visual deprivation, especially from birth on dynamic gait stability.

**Methods:**

The current study was conducted on 20 congenital blind and 10 sighted people (15–38 years). To evaluate the dynamic stability, descriptive data, harmonic ratio (HR), improved harmonic ratio (iHR), and root mean square (RMS), based on trunk acceleration data were measured in three axes: anteroposterior (AP), vertical (V), and mediolateral (ML) while participants walked an eight-meter straight path.

**Results:**

In the comparison of blind and sighted people (eyes open), standard deviation, HR, iHR, and RMS indices were found to be significantly different in both AP and V directions. All the mentioned parameters were significantly lower in blind than in sighted participants. In the comparison of blind people and sighted ones with closed eyes, changes were observed in the maximum, range, standard deviation, and RMS only in the AP axis. In the comparison between eyes open and closed in sighted people, a significant difference was found only in the harmonic ratio of the vertical axis.

**Conclusion:**

Visual deprivation led to a decrease in dynamic stability parameters in the AP and V axes. Even the movement of sighted people in unchallenged conditions is dependent on visual information.

## Introduction

The balance and control of the center of gravity of the body while walking is one of the main human movement behaviors [1]. Human upright standing is intrinsically unstable, and the central nervous system maintains balance by altering posture to maintain the center of gravity within the base of support [2]. Dynamic stability can be defined as the ability to control the position and momentum of the whole body during walking or other activities in which the center of gravity passes outside the base of support [3].

Dynamic stability helps the body move at a suitable speed and minimizes oscillations of upper body segments, thereby reducing the risk of falling [4]. Decreased dynamic stability increases the risk of falling [5]. It relies on multiple sensory inputs, including visual, somatosensory, and vestibular inputs, as well as spinal reflexes and cortically controlled gait patterns [6]. Disruption in the performance of controllers in the phases of sensing, processing, and command execution may lead to loss of standing balance and falls, which bring unknown consequences [7].

Compared to other sensory systems, vision has a major role to play in controlling human movement and balance [8]. Due to the importance of vision in walking and the fact that 80% of the information in the motion control system is obtained through vision, blind and severely visually impaired people usually experience changes in their gait pattern [9, 10]. These alterations can generally limit movement and physical activity, exposing people to numerous movement problems, such as falls [8].

According to the World Health Organization, falls are the second leading cause of unintentional injury death, claiming the lives of 684,000 people every year, 80% of which occur in developing countries [11]. Although falls have a multifactorial etiology, a commonly cited cause of falls is poor vision. Studies have pointed out that blind and partially sighted people are 1.9 times more likely to fall than their sighted peers due to a failure to adapt the environment to their needs and the lack of visual keys [12].

Therefore, the assessment of gait in blind people and dynamic stability as a quantitative method provides a golden opportunity to study the effects of this vital sensory channel on the recognition of the mechanisms involved in this movement skill. Moreover, in the design of assistive devices, their balance maintenance and control need to be taken into consideration. Different metrics are used to measure dynamic stability. In recent years, accelerometers have been introduced as a non-invasive, small, relatively low-cost, and practical alternative that can monitor human movement in different spaces [13].

Different parameters obtained from the accelerometer, including harmonic ratio, root mean square, accelerometer descriptive information, and entropy, have been referred to in different studies as indicators of dynamic stability [4, 14]. Most of the studies that have been conducted so far have investigated the effect of visual deprivation on the kinetic and kinematic parameters of walking [9, 10]. Nonetheless, there is a little information on the effects of visual deprivation on the dynamic stability of gait [4, 15]. For instance, Iosa et al. used accelerometers to investigate the effect of visual impairment on the dynamic stability of sighted (eyes open and closed) people [4].

To assess the dynamic stability of blind people, Hallman et al. analyzed the biomechanical walking patterns using the Vicon motion analysis system [16]. Today, the rehabilitation of people with physical disabilities has received assiduous attention, and this type of evaluation, especially using practical and less expensive equipment, can be of great help in the improvement of movement exercises, achievement of more independence, and development of rehabilitation strategies [17, 18]. Therefore, it is most important to identify the influential factors affecting walking in blind people to prevent the occurrence and reduce their movement disorders. Considering the limited studies on dynamic stability in visual deprivation, therefore, the present study was carried out to assess the dynamic stability of blind people compared to their sighted peers.

## Materials and methods

### Participants

This case-control study was carried out on 20 congenital blind individuals as a sample group and 10 sighted individuals as the controls. Blind individuals in the age range of 15–38 years whose blindness was approved by ophthalmology team in the Iranian Welfare Organization and had no other disabilities other than visual impairment were included in the study. All the participants in the blind group had congenital eye diseases, and the main cause of their blindness was primary congenital glaucoma (PCG) and congenital cataract. Their visual acuity was without light perception (11 people) and light perception (9 people). During the experiments, blindfolds were used to make the conditions the same. Normal controls were graduate students and staff members at the University of Medical Science, who had normal or corrected to normal visual acuity of 0.00 log MAR. Participants were excluded from the study in case of their unwillingness to cooperate and a history of surgery or injury in the lower limbs and spine. All participants provided written informed consent as per the ethics committee of the Shiraz University of Medical Science guidelines and the declaration of Helsinki.

### Data collection

After the participants (blind and sighted) entered the laboratory, their demographic characteristics, such as height, weight, and hand grip strength (both hands), were measured and recorded. Moreover, the subjects were asked to attend the laboratory in shoes they were comfortable with, except sandals and high heels. Following that, they were asked to walk around the laboratory to get familiar with the environment.

In this study, a three-axis accelerometer (ADXL345) and a microcontroller (AVR Atmega328) were used to collect data. The sampling frequency was 100 Hz sent to the mobile phone via Bluetooth and stored in a memory card. This accelerometer was attached to a belt along the anterior-posterior, vertical, and mediolateral axes close to the center of gravity of the body, around the third and fourth lumbar vertebrae to measure the desired factors. This position was selected due to its proximity to the body’s center of mass when standing. To control and not interfere with one’s movement, the accelerometer data recording application was designed and installed on the mobile phone to record the data.

The subjects in blind group walked 8-meter straight path at a preferred speed three times. The subjects in the sighted group walked 8-meter straight path in open and closed eye conditions at a preferred speed while looking at a fixed point at the eye level on the wall, three times in each condition.

**Fig. 1:**
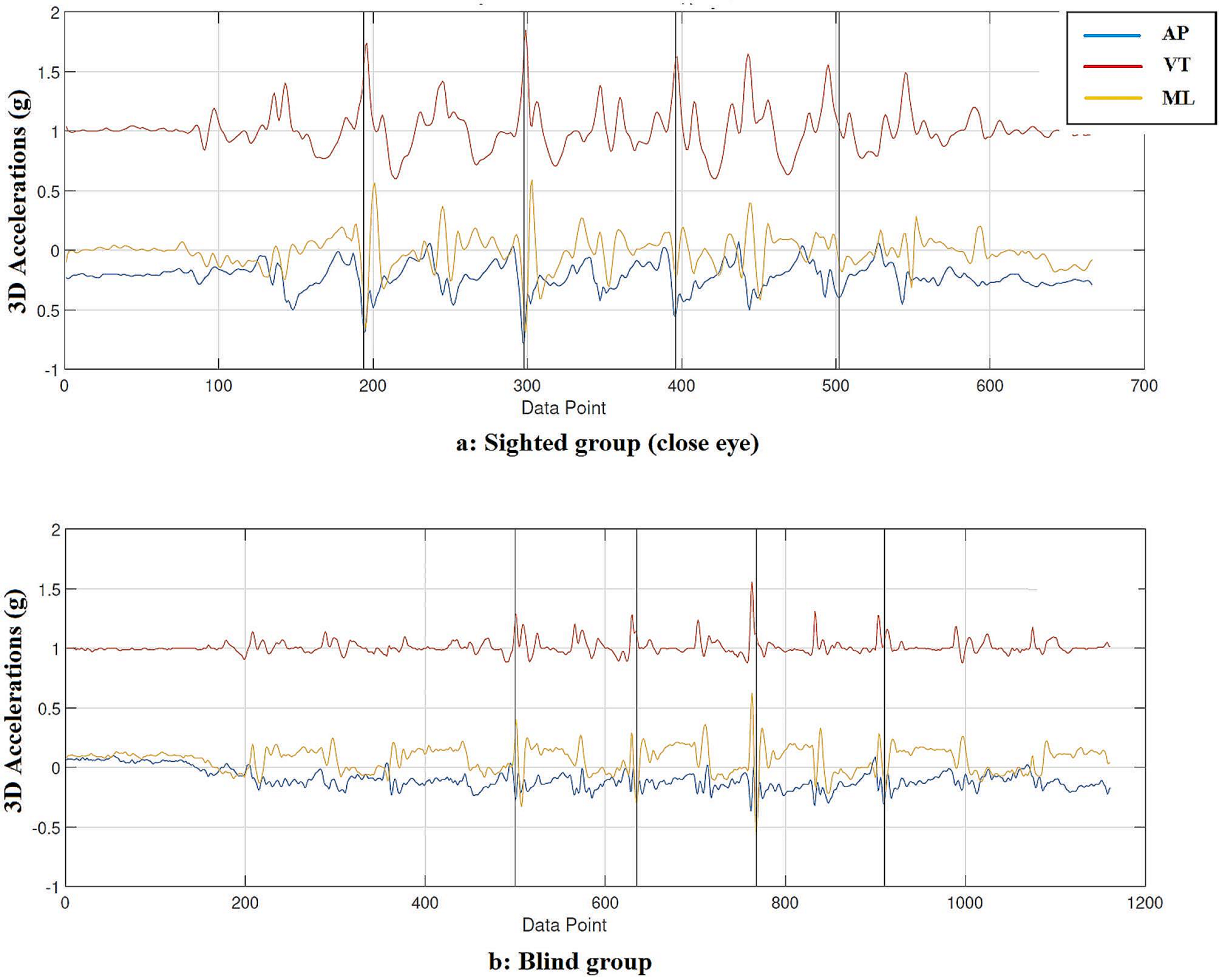
Schematic representation of gait asymmetry assessment: the three axis accelerometer and a recorded signal along anteroposterior (AP, blue line), vertical (VT, red line), and mediolateral (ML, yellow line) in sighted with open eye condition (**a**) and blind (**b**) group

In blind and sighted group, the first walking trial in each condition was considered to be practice and not used in the analysis. Considering that the gait pattern is different at the beginning and end of the path, three strides (three gait cycle) in the middle of the path in the second or third trial were considered for gait analysis according to the Fig. 1.

#### Data processing

Accelerometer signals were processed using MATLAB software (Mathworks Inc., USA).

Each stride (A gait cycle) is defined as the time from the first heel strike to the next strike of the ipsilateral heel. In the MATLAB software, the stride were separated based on peakfinder (Fig. 1). To filter the data obtained from the accelerometer, a bidirectional Butterworth filter with a cutoff frequency of 30 Hz was used [19]. Based on the studies conducted, the cutoff frequency of 30 and 40 Hz was confirmed as the best frequencies in maintaining the peak values and also removing the low values, and in this study, the frequency of 30 Hz was selected [20]. Then the parameters related to descriptive statistics, Root mean square and symmetry (HR and iHR) were calculated for each stride and then the average of all three stride was obtained.

##### Descriptive statistics

Minimum, maximum, range, mean, and standard deviation (g) obtained from the accelerometer in V, AP, and ML axes.

##### Gait speed

It is the time required to walk a certain distance (m/s). In this study, the gait speed was obtained by dividing the eight-meter travel path by the time spent moving [21].

##### Harmonic ratio

Harmonic ratio (HR) which indicates the degree of symmetry of two steps in a stride, is most commonly extracted from trunk accelerations in the anteroposterior (AP), vertical (VT), and mediolateral (ML) directions [12]. The harmonics resulting from Fourier analysis of each step were used to calculate the HR of walking. The harmonic ratio in the AP and VT axes is computed as the sum of the amplitudes of the even harmonics divided by the sum of the amplitudes of odd harmonics, and it is the opposite in the ML direction. An increase in the harmonic ratio can indicate an increase in order and symmetry [12].

$$ {HR}_{AP-V}=\frac{\sum {A}_{even harmonics}}{\sum {A}_{odd harmonics}}$$

12 $$ {HR}_{ML}=\frac{\sum {A}_{odd harmonics}}{\sum {A}_{even harmonics}}$$

##### Improved harmonic ratio

The improved harmonic ratio (iHR) derived from Fourier analysis of trunk accelerations is calculated by dividing the square of the even harmonics (AP and V directions) or odd harmonics (ML direction) by the sum of the squares of even and odd harmonics and then multiplying by 100 [22]. Therefore, this index varies from zero (total asymmetry) to 100 (total symmetry), which is easier to interpret and understand compared to the harmonic ratio [22].

$$ {iHR}_{AP-V}=\frac{\sum {\left({A}_{even harmonics}\right)}^{2}}{\sum {\left({A}_{even harmonics}\right)}^{2}+ \sum {\left({A}_{odd harmonics}\right)}^{2}}\cdot 100$$

22 $$ {iHR}_{ML}=\frac{\sum {\left({A}_{odd harmonics}\right)}^{2}}{\sum {\left({A}_{even harmonics}\right)}^{2}+ \sum {\left({A}_{odd harmonics}\right)}^{2}}\cdot 100$$

##### Root mean square

The Root Mean Square (RMS) is an indicator of dispersion of the data relative to zero, as opposed to the SD, which is a measure of dispersion relative to the mean [23]. The root mean square (RMS) of trunk acceleration is frequently used in gait analysis research [24]. The RMS calculation is very simple and does not require any preconditions, such as an optimal threshold. Therefore, the physical meaning of an RMS value is clear, and its increase is associated with an increase in speed [23].

### Statistical analysis

After collecting the information and entering the data into SPSS software, the data were analyzed using descriptive and analytical statistics. The Kolmogorov-Smirnov test was used to measure the normality of the data. Considering the normality of data distribution, the independent t-test was used to compare the two groups. The Chi-square test was also used to compare qualitative variables between the two groups. The significance level was set at 0.05 in this study.

## Results

Table 1 displays the demographic characteristics of participants in the two groups. As illustrated in this table, there was no significant difference between the demographic characteristics of participants in the two groups.

**Table 1:** Subject Characteristics in sighted and blind groups

Variables		Sighted person (*n* = 10)	Blind person (*n* = 20)	P value
Age (years)		28.10 (7.17)	24.91 (5.39)	0.165*
Height (cm)		163.10 (7.83)	163.92 (9.58)	0.814*
Weight (kg)		56.90 (9.27)	58.41 (11.94)	0.723*
BMI (kg/m^2^)		21.24 (2.46)	21.94 (4.45)	0.642*
Grip strength (Right hand) (kg)		31.30 (12.90)	28.68 (6.76)	0.443*
Grip strength (Left hand) (kg)		28.82 (9.93)	27.64 (7.74)	0.713*
Sex (male) (%)		50% (*n* = 5)	54.2% (*n* = 13)	0.452†
Marital status (single)		70% (*n* = 7)	79.2 (*n* = 19)	0.566†
Level of education	Diploma & lower	20% (*n* = 2)	37.5 (*n* = 9)	0.608†
	BSc	60% (*n* = 6)	45.8 (*n* = 11)	
	MS & PhD	20% (*n* = 2)	16.7 (*n* = 4)	
Dominant hand	Right	80% (*n* = 8)	79.2 (*n* = 19)	0.403†
	Left	20% (*n* = 2)	20.8 (*n* = 5)	

Values are means ± standard deviation or percentage. P values were calculated using independent t-test* or Chi-Square tests†

The investigated variables between the sighted and blind groups are presented in Table 2. In the comparison of blind and sighted subjects, most alterations were observed in the AP and V axes. In the comparison of the groups, no significant difference was observed in the ML axis. The comparison between eyes-closed and eyes-open states pointed to a significant relationship in the speed and harmonic ratio of the vertical axis.

**Table 2:** comparison of asymmetry gait variable between the blind and the sighted groups in direct path

Variables	Sighted (Mean + SD)		Blindness	P- value		
	Open eye	Close eye		Open vs. close eye	Open eye vs. blind	Close eye vs. blind
Walking speed (m/s)	1.04 (0.22)	0.82 (0.17)	0.65 (0.19)	0.048*	0.001**	0.071
***Acceleration descriptive statistics (g)***						
AP minimum	-0.34 (0.17)	-0.24 (0.16)	-0.20(0.23)	0.216	0.086	0.565
AP maximum	0.47 (0.21)	0.43 (0.17)	0.25 (0.15)	0.599	0.002^**^	0.008**
AP range	0.82 (0.32)	0.67 (0.25)	0.45 (0.16)	0.284	0.001**	0.005**
AP mean	0.09 (0.12)	0.10 (0.10)	0.05 (0.16)	0.985	0.470	0.446
AP standard deviation	0.19 (0.08)	0.15 (0.05)	0.10 (0.05)	0.250	0.001**	0.001**
V minimum	0.01 (1.08)	0.31 (0.97)	0.61 (0.58)	0.533	0.047*	0.278
V maximum	0.79 (1.02)	0.92 (0.85)	1.17 (0.60)	0.746	0.177	0.340
V range	0.77 (0.19)	0.61 (0.17)	0.56 (0.20)	0.077	0.011*	0.514
V mean	0.36 (0.94)	0.58 (0.83)	0.82 (0.55)	0.603	0.088	0.322
V standard deviation	0.17 (0.17)	0.14 (0.04)	0.12 (0.05)	0.193	0.016*	0.307
ML minimum	-0.24 (0.15)	-0.10 (0.36)	-0.18(0.05)	0.281	0.238	0.372
ML maximum	0.28 (0.11)	0.36 (0.29)	0.23 (0.13)	0.430	0.343	0.086
ML range	0.52 (0.23)	0.47 (0.13)	0.42 (0.16)	0.511	0.132	0.390
ML mean	0.02 (0.07)	0.14 (0.30)	0.03 (0.09)	0.279	0.887	0.130
ML standard deviation	0.11 (0.04)	0.10 (0.02)	0.09 (0.02)	0.451	0.085	0.351
***Harmonic Ratio (%)***						
HR-AP	2.22 (0.46)	2.20 (0.70)	1.82 (0.52)	0.941	0.043*	0.093
HR-VT	2.72 (0.50)	2.15 (0.56)	1.86 (0.51)	0.030*	0.001**	0.146
HR-ML	1.92 (0.41)	1.71 (0.38)	1.79 (0.44)	0.254	0.419	0.624
***Improved Harmonic Ratio***						
iHR-AP	83.47 (7.72)	82.11(10.69)	76.61 (9.24)	0.748	0.038*	0.140
iHR-VT	87.23 (7.64)	81.29 (9.38)	76.80 (10.96)	0.138	0.004**	0.265
iHR-ML	77.96 (8.34)	73.60 (6.68)	73.80 (10.35)	0.213	0.232	0.955
***Root mean square***						
RMS -AP	2.09 (0.85)	1.68 (0.51)	1.18 (0.35)	0.208	0.008**	0.002**
RMS-VT	1.87 (0.46)	1.63 (0.53)	1.43 (0.56)	0.278	0.028*	0.367
RMS-ML	1.34 (0.42)	1.18 (0.31)	1.09 (0.35)	0.341	0.125	0.508

Values are means ± standard deviation. P values were calculated using independent t-test

^*^Statistically significant at *p* < 0.05

** Statistically significant at *p* < 0.001

## Disscussion

The current study was conducted to investigate the dynamic stability of the blind compared to sighted people with their eyes open and closed.

1- Comparing blind and sighted people (while having their eyes open): The highest amount of changes in the parameters related to dynamic stability between the sighted and blind groups were observed in the AP and V axes. In this regard, a significant difference was found among the average factors of speed, range, standard deviation, HR, iHR and RMS in both the AP and V axes, and between sighted and blind people. However, no significant difference was noticed in the ML axis comparing these two groups.

The results of the present study indicated that amongst the investigated variables, the speed of gait in blind people is almost 63% lower than the speed in sighted people. According to the conducted studies, slowing down can be the result of a cautious motor mechanism, which is due to the elimination of the anticipatory mechanism and the lack of environmental input through the visual system [9, 25]. On the other hand, the reduction of gait speed in the absence of vision for maintaining the stability of the body position gives the blind extra time to have a more appropriate and more balanced response, especially through their sense of touch [26]; this can be seen in reducing the standard deviation of the gait pattern of blind people compared to sighted people. In this analysis, the standard deviation of AP and V axes in blind people was 0.10 and 0.12 (g), respectively, and these figures were 0.19 and 0.17 (g) for sighted people.

Based on what is stated in Table 2, the range of accelerometer data in the AP and V axes was 0.82 and 0.77 (g) in the sighted group and 0.45 and 0.56 (g) in the blind group, respectively. Larger values of descriptive data associated with acceleration may reflect a more consistent gait pattern with less range of variation [27]. In this regard, the increase in descriptive parameters related to acceleration in the AP and V axes can indicate an increase in parameters related to balance.

HR and iHR are indices of symmetry, and the reduction of these two indices illustrates the decrease of symmetry and increase of irregularity in steps. The results of the present study showed that in blind people’s group, HR in the AP axis decreased by 0.4% and in the vertical axis by 0.86% compared to sighted people’s group. According to Table 2, the amount of iHR in the AP and V axes was significantly reduced in the group of blind people. The results of this study indicate that the presence of vision results in a more symmetrical performance in steps; according to these findings, irregularity in walking and asymmetry can cause a decrease in dynamic stability and an increase in the probability of falling [4, 5]. In the study conducted by Majlesi et al., the symmetry index was investigated in the parameters of stepping and the comparison of the left and right legs in sighted and blind people, and no significant difference was found between the two groups [10]. The dissimilarity in the results of these two studies may be due to the difference in the indices measured for symmetry.

In this study, the RMS in the AP axis in the sighted and blind groups was calculated as 2.09 and 1.18, and in the V axis, as 1.87 and 1.43. A significant decrease in RMS in blind people can be the result of a decrease in walking speed in these people. Several studies have shown that RMS is affected by walking speed and balance, and has a direct relationship with these factors; and this difference can be seen in all three anatomical axes [10, 28]. In general, the results of this part of the study are in line with the findings of Huijben et al. who worked on a population of elderly people and realized that an increase in walking speed will lead to higher step frequency, higher standard deviation, more symmetrical walking and generally a more stable walking pattern [29].

2- Comparing blind and sighted people (with their eyes closed): A significant difference was seen in the variables of maximum, range, standard deviation and RMS value in the AP axis between the two groups.

The standard deviation of the AP axis was calculated as 0.15 (g) when a sighted person moved with their eyes closed and 0.10 (g) for a blind person. An increase in the standard deviation in the sighted group can indicate a disturbance in the balance and displacement of the body’s center of gravity. Due to having enough time to create a more appropriate response, blind people slow down their walking speed to maintain postural stability and this can be seen in reducing the standard deviation [26].

The maximum variables and the range of accelerometer data in the AP axis were 0.43 and 0.67 (g) in the sighted group while having their eyes closed, and 0.25 and 0.45 (g) in the blind group, respectively. Larger values of descriptive data associated with acceleration could reflect a more consistent walking pattern with less range of variation [27].

Based on the results of this study, no significant difference was found in the walking speed of blind and sighted people with closed eyes. Therefore, the reason for not changing the symmetry may be due to the relative stability of the walking speed.

The root mean square in the AP axis was calculated for sighted people (with their eyes closed) as 1.68, and 1.18 for blind people. This parameter indicates the magnitude of acceleration; and several studies have shown that RMS is affected by walking speed and balance [30]. In this part of the study, RMS showed a meaningful decrease in blind people. In this regard, the authors did not find a study that examines the mentioned variables comparing blind and sighted people with their eyes closed.

3- Comparing the conditions of movement with eyes open and closed in the group of sighted people: Although all the variables decreased while the eyes were closed, a significant difference was found only in the walking speed and the harmonic ratio of the vertical axis.

The walking speed decreased from 1.04 m/s with their eyes open to 0.82 m/s while their eyes were closed. Due to the reliance on visual information, sighted people showed significant changes in movement speed to maintain the walking pattern.

HR in the vertical axis was 2.72% when their eyes were open and 2.15% when their eyes were closed. These results illustrate that in case of deprivation of visual feedback, the symmetry in the vertical axis can be reduced. These changes in sighted people can be due to a lack of adaptation to these conditions. In the study of Iosa et al., only sighted people participated to compare vision effect, and several other parameters such as RMS and HR were utilized to check the stability [4]. The results of this study indicated a decrease in the RMS index in all three axes, and HR only in the AP axis. In Majlesi study, there was no significant difference in speed comparing these two modes [10].

The findings of our study and other studies demonstrate that vision plays a crucial role in the development and maintenance of balance, and balance is reduced because of a lack of vision [31, 32]. Therefore, balance analysis in blind people can help to identify problems related to it, including the state of balance in different axes of the body, to develop strategies to prevent possible events, rehabilitate walking, especially in terms of Orientation and mobility (O&M) training among these people [33].

## Conclusion

As evidenced by the results of this study, vision can affect dynamic parameters. Visual deprivation led to a decrease in dynamic stability parameters investigated in this study in the AP and V axes. Even the movement of sighted people in unchallenged conditions is dependent on visual information.

## Recommendations

It is suggested to assess the effect of assistive devices on orientation and mobility, as well as different environments and their impact on dynamic stability.

## Data Availability

The datasets used and/or analyses during the current study are available from the corresponding author on reasonable request.
